# 

**Published:** 1895-01

**Authors:** 


### ILLUSTRATIONS.

Apparatus for administering chloroform to horses . . . . . . 657

Apparatus for irrigating purposes in epizootic abortion in cattle . . 472

Anatomy of the large American fluke . . . . 141, 145, 218, 220

An improved veterinary thermo-cautery . . . . . . 602

Gamgee, Joseph . . . . . . . . . . . 319

Horseshoeing . . . . . . . . . . 271, 275

New York College of Veterinary Surgeons:

Canine hospital . . . . . . . . . . 410

Chemical laboratory . . . . . . . . . 409

Hospital . . . . . . . . . . . 406

Pasteur, Louis M. . . . . . . . . . . . 749

### INDEX.

ABDOMINALIS, paracentesis, 649
Abortion, epizöotic, outbreak of, 469
Acheson, H. W., 331
Acid, iodic, treatment of tumor with, 668
Actinomycosis, 487
Action of rattlesnake venom upon the bactericidal power of blood-serum, 170
Adamson, J. H., 148
Address at the Commencement of the New York College of Veterinary Surgeons, 201
 to McGill graduating class, 298
 President's, before the U.S.V.M.A., 708
  before the Missouri Valley Veterinary Association, 497
  California Veterinary Association, 301
  Virginia State Veterinary Association, 561
Affections of the heart and acute founder in the horse, 356
Allen, F. S., 696
Alimentary canal, foreign matter in, 742
 tuberculosis infection through, 435
Alverson, A. G., 176
American beef-products, embargoes against, 181
 cattle, prohibition of importation of, 42
 horseflesh in European food-markets, 321
 Institute Farmers' Club, 52
 sheep-disease, so-called, 372
 Veterinary College, 241, 611
  Association, 194
  Alumni Association, annual meeting, 260
  Pennsylvania Alumni Association of, 191, 260
  reunion of graduates, 380
 Review, editorial in, 599
 journalism, what is the matter with, 518
Among people and their doings, 773
 veterinarians, marriages, 459, 524, 780
 the Colleges:
  American Veterinary College, 611
  British Veterinary Schools, 771, 853
  Chicago Veterinary College, 611
  Detroit College of Medicine, veterinary department, 610
  Harvard Veterinary Department, 770
  Indiana Veterinary College, 451
  Kansas City Veterinary College, 448
  McGill University, Veterinary Department, 517
  McKillip Veterinary College, 449
  National Veterinary College, 610
  New York College of Veterinary Surgeons, 517
  Ohio State University, Veterinary Department, 447, 770
  Ohio Veterinary College, 609
  Ontario Veterinary College, 609
  Royal College of Veterinary Surgeons, 450
  University of California, 769
   of Pennsylvania, Veterinary Department, 450, 516
Anatomy of the large American fluke (fasciola magna) and a comparison with other species of the genus fasciola, S. St., 139, 213, 277
Animals, reminiscence of intelligence of, 731
Animal vaccination, 601
Anthrax control-work, 673
Antiseptic surgery, 283
Antitoxin, 228, 369
 diphtheria, production of, 229
 therapeutic use of, 233
Anus, imperforate, 580
Archibald, R. A., 57, 257, 464
  people of the State of California *vs.*, 388
Arizona legislation, 309
Army legislation, U.S.V.M.A., report of committee on, 771
Art of farriery, progress in, 196
Arytenectomy for roaring in the horse, 823
Association duty of the hour, 841
 Guernsey Breeders', 250
 meetings, 598
 of Veterinary Faculties, 315, 374, 526, 685
Attitude of Massachusetts Farmers' League, 746
Autopsies, 393, 577, 578
Azoturia, treatment for, 110

BABB, Albert, 331
Bactericidal power of blood-serum, action of rattlesnake venom upon, 170
Barth, W. C., 487
Beef-products, American, embargoes against, 181
Belladonna, 431
Benjamin, M. H., 663
Bishop, S. P., 431
Bitch, enchondroma in, 222
 enterotomy on, 740
Black, Judson, 742
Bland, Charles, 743
Board of Examiners, New York's, 683
Board, State, of Veterinary Examiners, New York, 597, 667
  Pennsylvania, 739
 of Trade, Minneapolis, milk- and meat-inspection meeting, 369
Boutelle, C. A., 222
Brain, tumors of, disclosed by autopsy, 502
British veterinary schools, 771
Browning, G. W., 817
Busman, H., 288

CALIFORNIA, State Veterinarian for, 36
 veterinary legislation in, 43
 State Veterinary Association, 45, 253, 462
  synopsis of President's Address, 301
 the people of the State of, *vs.* Dr. R. A. Archibald, 388
 University of, 769
Cancer and tuberculosis, the pilocarpine treatment of, 325
Candidates, examination of, State of Ohio, 755
Canine distemper, 92
Caninus, gastritis, 697
Carpenter, Tom, 591
Castration of cryptorchids, 817
 reminiscences of, 492
Cataract operations on a dog, 133
Cat, defective development of central nervous system in, 393
 show, the national, 318

Cattle fever investigations, 427
outbreak of epizoötic abortion in, 469
new cause of death of, 477
poisoning of, with nitrate of sodium, 78
selection in, as a preventive of diseases among people, 490
Celebration, the Rudolph Leuckart, 735
Changes in the personnel of the Bureau of Animal Industry, 387
Chicago Veterinary College, 310, 611
Chloroform, administration of, to horses, improved apparatus for, 656
Choate, H. H., 130
Civil-service examination, New York City, 755
Clark, W. G., 259
Claussen, W. R., 733
Clement, A. W., 104, 444, 671, 829, 838
Clinical gleanings, 386, 446
Clitoris, hypertrophy of the, 671
Closing days of our college courses, 182
Coal-tar and some of its medicinal products, 162
Colic, flatulent, 302
therapeutics of, 613
College, the United States and the three-year course, 319
Colt, operation for umbilical hernia in, 104
Columns, veterinary in secular journals, 317
Commencement exercises:
American Veterinary College, 241
Ames, Veterinary Department, 853
Chicago Veterinary College, 310
Detroit College of Medicine, Veterinary Department, 672
Harvard Veterinary Department, 516
Indiana Veterinary College, 853
Kansas City Veterinary College, 240
McGill University, Veterinary Department, 311
National Veterinary College, 313
New York College of Veterinary Surgeons, 242
Ohio State University, Veterinary Department, 446
Ontario Veterinary College, 236
University of Pennsylvania, Veterinary Department, 446
United States College of Veterinary Surgeons, 313
Commission, tuberculosis, 115
Complication, septicæmia following parturition, 578
Congress, Berne, International Veterinary, 328
Contagious pleuro-pneumonia, groundless reports of, 316
Control of tuberculosis in Massachusetts, 791
work, California, 833
Australian Colonies and New Zealand, 744
Illinois, 673
Iowa, 745, 834
Indiana, 834
Louisiana, 745
Massachusetts, 672, 673, 745, 835
Minnesota, 673
New York, 673, 835
Oregon, 836
Pennsylvania, 673, 835
Tennessee, 673
Vermont, 673
Wisconsin, 836
Convention, echoes of, 675
of college faculties, 374, 526, 685
Correction, 44
Cows, non-tuberculous, experiments with tuberculin on, 537
Croupous pneumonia in the horse, 155
Crowforth, A., 20

Dairies, inspection of, 509
Dealing with tuberculosis, uniform movement for, 673

Degree or title, necessity for uniform, 112
Delay, apology for, 374
Dell, Harri H., 768
Dermoid cyst, 393
Des Moines, on to, 452
Desmond, veterinary surgeon, 469
Diagnosis, physical, 640, 711
Diphtheria antitoxin, the production of, 229
toxin, the production of, 228
Distemper, canine, 92
in dogs, 292
District of Columbia Veterinary Medical Association, 331
Dixon, Marshall and, 582
Dog, intussusception in a, 394
hæmatosalpinx and hæmatometra in a, 577
pyosalpinx in a, 577
two successful cataract operations on, 133
Dogs, distemper in, 292
Duane, John, Jr., obituary, 847
Duncan, J. T., 580

Echoes of the convention, 675
Editor and owner, our new associate, 371
Editorials:
A more confident public gradually developing, 454
And now Pennsylvania, 746
Animal vaccination, 601
Apology for delay, 374
Association, duty of the hour, 841
meetings, 598
of veterinary faculties of North America, 315, 374, 685
Attitude of the Massachusetts Farmers' League, 746
Closing days of our college courses, 182
Contagious pleuro-pneumonia, groundless report of, 316
Convention of college faculties, 518
Editorials in *American Veterinary Review*, 1895, 599
Embargoes against American beef-products, 181
Error, 113
Facts, not forebodings, 679
Good roads, 371
Groundless report of contagious pleuro-pneumonia, 316
Growth of veterinary literature, 372
Important notice, 181, 246
Is this a blunder? 845
Journal change, 844
Kansas City Veterinary College, 748
Laymen in the Department of Agriculture in England, 747
McKillip Veterinary College, 684
Missing Journals, 181, 246
National cat show, 318
Necessity for a uniform degree or title, 112
Notice to our readers, 518
New York's Board of Examiners, 683
desires a higher standard, 112
State Veterinary Association, 372
On to Des Moines, 452
Opposition to State examiners in Ohio, 315
Our December number, 844
Our friend Bryden, 520
Our journey westward, 686
One more word, 843
Our new associate editor and owner, 371
Pennsylvania points the way, 373
State Board of Veterinary Examiners, 748
Prof. Theobald Smith, 747
Prohibition of importation of American cattle, 42
Prospectus of Journal, 1896, 840
So-called "American sheep-disease," 372
State Board of Veterinary Examiners, 315
The closing days of our college courses, 182
The new outlook, 519

Editorials (*continued*):
To our contributors and readers, 601
To our subscribers, 374
United States College and the three-years' course, 319
Veterinary Medical Association, 375, 682
a national charter for, 44
Uniform veterinary degree, 598
Veterinary columns in secular journals, 317
legislation in California, 43
in New York, 112
meat-inspection, 846
What is the matter with American veterinary journalism? 518
Wisconsin State meeting, 519
Educational, 183, 298, 370, 460
veterinary, in relation to the practice of veterinary medicine in New York State, 1
Embargoes against American beef products, 181
Enchondroma in the bitch, 222
Enlargement of the near hock, 829
Enteralgia, 721
Enterotomy on a bitch, 740
Epizoötic abortion, outbreak of, in cattle, 469
sore mouth of sheep in Montana, 412
Equine foods, 59
hermaphrodite, 391
pneumonia, morbid histology and bacteriology of, 421
Eradication of tuberculosis by a practical and profitable method, 474, 573, 646
Ernst, John, 490
Eruptic fever, 9
Essence of turpentine, subcutaneous injections in pneumonia, 355
European food markets, American horseflesh in, 321
Every-day points in veterinary jurisprudence, 496
Ewing, Charles B., 170
Examination Questions:
Veterinary, State of New York, 752
Civil-service examination, New York City, 755
Candidates' examination, State of Ohio, 755
Experiments with tuberculin on non-tuberculous cows, 537
Extinction of tuberculosis, 808
Extracts from foreign journals, 827
Extremities, diseases of, immobility in the treatment of, 703

FACULTIES of Veterinary Colleges of North America, Association of, 315, 374, 518
Facts and acts worth knowing, 394
Farmers' League, Massachusetts, attitude of, 746
Farriery, progress in the art of, 196
Faville, George C., 128
Feiser, R. P., 392
Fever, Southern cattle, 78
Ferster, J. H., 348
Flatulent colic, 302
Fluke, anatomy of large American, 139, 213, 277
Foods, equine, 59
Food, horseflesh for our, 65
-markets, European, American horseflesh in, 321
Found among many places and sources, 854
Foreign veterinarians in tuberculosis, work of, 797
Founder, acute, and affections of the heart, 356
Fracture of the trapezium, 582
France, legislation in, 382
French, Cecil, 421, 697, 740

GAMGEE, Joseph, 319
GAMGEE, John, 247
Gastritis, caninus (non-specific), 697
German Veterinary Association of New York, 465
Gill, H D., 229, 393, 502, 653, 804
Glanders, 360
and mallein, 653
Gleanings, clinical, 386, 446
Good roads, 371
Goss, Eugene, 391
Gossip, 381
Graduates of American Veterinary College, reunion of, 380
Growth of veterinary literature, 372
Guernsey Breeders' Association, 250, 423

HÆMATOSALPINX and hæmatometra in a dog, 577
Hamilton, F. A., 427
Harbaugh, W. H., 78, 561, 632, 717
Harger, S. J. J., 165, 823
Hassall, Albert, 213
Harvard Veterinary Department, 516, 770
Heart, affections of, and acute founder, 356
Heath, A. S., 474, 573, 646
Helmer, Jacob, 640, 711
Heiser, S., 392
Hermaphrodite, equine, 391
Hemorrhagica, purpura, 9
Hinkley, N. P., 124, 462, 531, 763
Histology, morbid, and bacteriology of equine pneumonia, 421
Hoare, E. Wallis, 443, 656, 707
Hock, enlargement of the near, 829
Honors to veterinary educators, 369
Hopper, J. B., 261
Horse, arytenectomy for roaring in, 823
-chestnut in pulmonary emphysema in the horse, 66
croupous pneumonia in, 155
Horseflesh, American, in European food-markets, 321
for our food, 65
"Horse industry" in Kansas, 361
Horseshoeing, 269, 348
Hoskins, W. Horace, 246, 304, 330, 443, 708
Huddleston, J. H., 233
Huff, Wilson, 726
Huidekoper, Rush Shippen, 65, 269
Hunter, S. L., 52, 260, 533
Hydrophobia, 148
Hygiene, 726
stable, 480, 733
Hyovertebrotomy, 147
Hypertrophy of the clitoris, 671

IMMOBILITY following influenza, 304
in diseases of extremities, 703
Imperforate anus with false opening, 580
notice, 181, 246
Importation of American cattle, prohibition of, 42
Improved apparatus for the administration of chloroform to horses, 656
Indiana Association of Veterinary Graduates, 690
Veterinary College, commencement exercises, 853
Infection, tuberculosis, through the alimentary canal, 435
Infectiousness of milk, 114
Influenza organism, 369
Injections, subcutaneous, of turpentine in pneumonia, 355
Injuries by trolley, temporary paralysis of peristalsis following, 443
Inspection of dairies, 509
meat, 71
Intelligence of animals, 731
Interesting post-mortems, 176
International Congress of Veterinary Medicine, Berne, Switzerland, 328

Inventions, new, 398, 468, 534, 778
Investigations concerning Texas cattle fever, 427
Iodic acid, treatment of tumor with, 668
Iowa State Veterinary Association, 608, 764
Is this a blunder, 845
Items of interest, 57, 267, 197
Illinois State Veterinary Medical Association, 331

JASME, A., 741
Jejunum, protrusion of, through the vagina, 392
Jopling, Wm., 132
JOURNAL change, 844
JOURNALS, missing, 181, 246
Journey westward, 686
Jurisprudence, veterinary, 496

KANSAS CITY VETERINARY COLLEGE, 240, 448
horse industry in, 361
legislation, 307
Keystone Veterinary Association, 49, 194, 257, 330, 379, 606, 696, 852
Klutts, L. M., 14
Knott, M. O. M., 578
Kidney, physiology of, 288

LAW, JAMES A., 201, 808
Laymen in department of agriculture in England, 747
League, Massachusetts Farmers', attitude of, 746
Legislation in Arizona, 309
California, 43
District of Columbia, 265
France, 382
Kansas, 307
Maryland, 384
Massachusetts, 264, 308, 383, 515, 593
Missouri, 308
National, 261
New York, 106, 112, 306, 384, 512, 737
Pennsylvania, 232, 307, 383
dehorning of cattle, 179
nicking and docking of horses' tails, 179
Lehnert, E. H., 188
Leithwood, Jas., 363
Lellman, Wilfried, 583, 668
Liautard, A., 1, 677
Literature, veterinary growth of, 372
Lockwood, S., 51, 332, 769
Lusson, L. O., 110

MAINE VETERINARY ASSOCIATION, 128
Mallein and glanders, 653
Marriages among veterinarians, 459, 524, 780
Martin, Luther, 43
Martenet, W. H., 292
Maryland legislation, 384
Veterinary Society, 130
Massachusetts, control of tuberculosis in, 791
legislation, 264, 308, 383, 515
Veterinary Association, 45, 47, 125, 184, 332, 849
Mayo, N. S., 336, 477
McEachran, D., 298
McGill Graduating Class, address to, 298
University, 195, 311, 517
McKillip, M. H., 638
Veterinary College, 449, 684
Mead, R. N., 412
Measures, sanitary, review of, 39
Meat and milk inspector, appeal for selection of a proper, 521
inspection in Germany, 423
inspection, 71
veterinary, 846
Michener, Isaiah, 117, 731
Michigan State Veterinary Association, 131
Milk, infectiousness of, 114
Mills, Wesley, 64
Minneapolis Board of Trade milk and meat inspection meeting, 370
Missing JOURNALS, 181, 246
Missouri State Veterinary Association, 607, 849
Valley Veterinary Association, 51, 259, 461, 532, 850, 853.
Montreal Veterinary Association, 185, 767, 851
Morbid histology and bacteriology of equine pneumonia, 421
Morris, Claude, 71
Morrow, F., 194

NATIONAL cat show, 318
legislation, 261
Veterinary College, 313, 610
Nerve. radialis, paralysis of, 501
Neurotomy, plantar, improved method of, 638
New and useful veterinary instruments, 467
cause of death of cattle, N. S. Mayo, 477
Hampshire State Veterinary Association, 381, 462
inventions, 197, 398, 468, 534, 778
Jersey, Veterinary Association of, 50, 332, 768
outlook, 519
York County Veterinary Association, 379
College of Veterinary Surgeons, 403, 517, 608
College of Veterinary Surgeons—commencement exercises, 242
German Veterinary Medical Society of, 465
College of Veterinary Surgeons, address at commencement of, 201
desires a higher standard, 112
legislation, 106, 112, 306, 384, 512, 737
State Veterinary Society, 118, 461, 757
Board of Medical Examiners, 597, 667, 683
veterinary examinations, 752
veterinary license law, 830
publications, 376
Niles, E. P., 99
W. B., 781
Noack, Otto, 302, 303, 423, 501, 521
Notes on tuberculosis, 99
Notice to our readers, 558

OBITUARY of Anderson, Louis A., 576
Autenreith, Joseph F., 576
Ardary, Frank, 43
Burchsted, George B., 392
Bryden, William, 527
Duane, John, Jr., 847
Gamgee, John, 247
Joseph, 319
Heiser, Samuel, 392
Martin, Luther, 43
McLean, R. A., 847
Pasteur, Louis, 748
Records, J. H., 576
Ohio State candidates' examination, 755
University Veterinary Department, commencement exercises, 446, 447, 470
Veterinary College, 400, 609
Oneida County Veterinary Society, 193
One aspect of new legislation, 385
Ontario Veterinary Association, 47
Western, 192
College, 236, 609
Society, 47
Operation for radical cure of umbilical hernia in a colt ten months old, 104
Opposition to State examiners in Ohio, 315
Organism, influenza, 369
Orvis, C. B., 301
Osgood, F. H., 791
Osteosarcoma in the nose of an ox, 393
Our December number, 844
Outbreak of epizoötic abortion in cattle, 469

PAIGE, James, 89
Paracentesis abdominalis, 649
Paralysis of the nerve radialis, 501
Park, William H., 228
Parker, John M., 46, 47, 127, 185, 195
Pasteur, Louis, 748
Pearson, Leonard, 797
Pennsylvania Alumni Association, American Veterinary College, 191, 606
legislation, 179, 232, 307, 383
points the way, 373
State Veterinary Association, 189, 608, 690
State Board of Veterinary Examiners, 739, 831
Peritonitis, 582
Personals, 244, 332, 401, 459, 524, 603, 689, 855
Personnel of the Bureau of Animal Industry, important changes in, 387
Peters, Austin, 337
Physical diagnosis, 640, 711
Physiology of the kidney, 288
Pilocarpine treatment of tuberculosis and cancer, 325
Pleuro-pneumonia, contagious, 316
in Kansas, 321, 326
Plural pregnancy, 632, 717
Points in practice, 628
in veterinary jurisprudence, 496
worth knowing, 268
Poisoning of cattle with nitrate of sodium, 89
Pope, Lemuel, 381, 462
Powell, N. C., 427
Pneumonia, bilateral, 302
croupous, 155
equine, 421
subcutaneous injections of turpentine in, 355
Preventive of disease among people, selection in cattle as a, 490
Priest, F., 412
Prohibition of importation of American cattle, 42
Proprietary remedies in veterinary practice, 441
Prospectus of JOURNAL, 1896, 840
Protrusion of the jejunum through the vagina, 392
Protestation, 677
Public, a more confident, 454
Pulmonary emphysema in the horse, the horse-chestnut in, 663
Purity of milk, 367
Purpura hemorrhagica, 9

QUITTORS, the use of pyoctanin in, 583

RADICAL cure of umbilical hernia in a colt, 104
Randolph, Robert L., 133
Rattlesnake venom, action of, upon the bactericidal power of blood-serum, 170
Rawle, Joseph, 496
Readers, notice to our, 518
Recto-vaginal fistula, 741
Recueil de Medicine Veterinarie, 356, 360, 667
Remarks on Southern cattle fever, 78
Remedies, proprietary in veterinary practice, 441
Reminiscences of castration, 492
of intelligence of animals, 731
Reports of cases:
An interesting case, 110, 670, 743
An obscrue case, 444
Anus vulvalis, 580
Autopsies, 577, 578
Bilateral pneumonia, 302
Enlargement of near hock, 829
Enterotomy on a bitch, 740
Flatulent colic, 302
Foreign matter in alimentary canal, 742
Fracture of the trapezium, 582
Hæmatosalpinx and hæmatometra in a dog, 577
Reports of cases (*continued*):
Hypertrophy of the clitoris, 671
Immobility following influenza, 304
Imperforate anus with false opening, 580
Paralysis of the nerve radialis, 501
temporary, of peristalsis following injuries, 443
Peritonitis, 582
pyoctanin, use of, in quittors, 583
Pyosalpinx in a dog, 577
Recto-vaginal fistula, 741
Treatment for azoturia, 110
of tumor with iodic acid, 668
Tumors of the brain disclosed by autopsy, 502
Unusual complication attending septicæmia following parturition, 578
Value of tips in shoeing, 743
Report of committee on Army Legislation, 771
on vital statistics, Virginia State Veterinary Association, 553
Reunion of graduates of American Veterinary College, 380
Reviews:
Antisepsis and Antiseptics, 248
Book on Cats, 320
Every Man His Own Horse Doctor, 41
Friedberger and Fröhner's Pathology and Therapeutics of the Domestic Animals, translated by Dr. W. L. Zuill, vol. i. 41; vol. ii, 247
Manual of Veterinary Micro-biology, 114
of Veterinary Therapeutics, 113
The Horse: its External and Internal Organization, 528
Translation of Moller's Operative Surgery, John A. W. Dollar, M.R.C.V.S., 348
Reynolds, M. H., 370
Rhoads, W. L., 50, 192, 194, 258, 331, 379, 381, 606, 696, 764
Roaring in the horse, arytenectomy for, 823
Royal College of Veterinary Surgeons, 450
Rodger, J. C., 690
Rudolph Leuckhart celebration, 735
Ryder, J. E., 330, 380

SALLADE, J. W., 9, 52, 480
Salinger, A. W., 155
Sanitary measures, regulation of veterinary, 39, 114, 115, 179
Schuylkill Valley Veterinary Association, 52
Selections, 36, 39, 106, 179, 250, 321, 325, 326, 355, 356, 361, 369, 435, 441, 521, 653, 656, 663, 667
Separator skim-milk, 321
Sheep in Montana, epizoötic sore mouth of, 412
Shoeing, value of tips in, 743
Smith, Theobald, Prof., 747
So-called "American" sheep disease, 372
Society proceedings:
American Institute Farmers' Club, 52
Veterinary College Alumni Association, 260
Veterinary Association of, 194
Annual meeting of the Oneida County Veterinary Society, 193
California State Veterinary Association, 45, 253
District of Columbia Veterinary Association, 331
German Veterinary Association of New York and vicinity, 465
Illinois State Association, 331
Indiana Association of Veterinary Graduates, 690, 853
International Veterinary Congress, Berne, Switzerland, 328
Iowa State Veterinary Association, 608, 764
Keystone Veterinary Association, 49, 194, 257, 330, 379, 606, 696, 763, 852
Maine Veterinary Association, 128, 381
Maryland Veterinary Society, 130

Society proceedings (*continued*):
Massachusetts Veterinary Association, 45, 125, 184, 332, 849
McGill University Alumni, 195
Michigan State Veterinary Association, 131
Missouri State Veterinary Association, 607, 849
Valley Veterinary Association, 51, 259, 461, 532, 850, 853
Montreal Veterinary Association, 185, 767, 851
New Hampshire Veterinary Association, 381
New York State Veterinary Society, 118, 461, 531, 608, 757
North Dakota Veterinary Association, 381
Ontario Veterinary Association, 47
College, 47
Pennsylvania Alumni Association of the American Veterinary College, 191, 606
State Veterinary Association, 189, 608, 690
Reunion of Graduates of the American Veterinary College, 380
Schuylkill Valley Veterinary Association, 52
United States Veterinary Association, 53, 453, 604
Veterinary Association, State of New Jersey, 50, 332, 768
Association of New York County, 330, 379
Virginia State Veterinary Association, 127 533
Western Ontario Veterinary Association, 192
Wisconsin Society of Veterinary graduates, 250, 609
Wyoming Valley Veterinary Association, 381, 607
Some interesting post-mortems, 176
Southern cattle-fever, 78, 427
Spencer, Sr., H. A., 283
Stable hygiene, 480, 733, 836, 837
State Board of Veterinary Examiners, Pennsylvania, 739, 831
New York, 667
State veterinarian for California, 36
Stewart, S., 497
Stiles, Charles W., 139, 213, 277, 586
Stringhalt, 162
Suggestive of a smile, 612
Suggestion or aid to the eradication of tuberculosis, 474, 573, 646
Surgery, antiseptic, 283
Sweetapple, C. M., 49

TETANUS, 503
Texas cattle-fever, concerning, 427
Therapeutics of colic, 613
Therapeutic use of antitoxin, 233
Title or degree, necessity for a uniform, 112, 248, 390, 457, 586, 598
Treacy, M J., 492
Treatment of diseases of extremities, immobility in, 703
for azoturia, 110
Toxin-diphtheria production, 228

Tuberculin, experiments with, on non-tuberculous cows, 537
Tuberculosis, 337, 672, 781
as viewed by a veterinarian, 804
commission, 115
control of, 791
extinction of, 808
in relation to animal industry and public health, 20
its prevalence and importance, 20
notes on, 99
suggestion or aid to the eradication of, 474, 573, 646
uniform movement for dealing with, 673
work of foreign veterinarians in, 797
Tuberculous infection through the alimentary canal, Sheridan Delapine in *Veterinary Record*, 435
Tumors of the brain, 502
treatment of, with iodic acid, 668
Turner, J. P., 773
Turpentine, subcutaneous injections of, 355

UNITED STATES COLLEGE and three-years' course 315
of Veterinary Surgeons, 310
Veterinary Association, 53, 334, 371, 399, 455, 604, 682, 771
national charter for, 44
University of California, 769
of Pennsylvania, 446, 450, 516

VACCINATION, animal, 601
Veterinary education in relation to the practice of veterinary medicine in New York State, 1
examinations, State of New York, 752
instruments, new and useful, 467
journalism, American, what is the matter with, 518
jurisprudence, 496
Meat-Inspection, Morton, 846
Medical Association, American Veterinary College, 194
of New Jersey, 332, 768
New York County, 330, 379
practise, proprietary remedies, 441
record, 441, 443, 663
thermo-cautery, an improved, 602
titles, 454
Virginia State Veterinary Medical Association, 127, 533

WADAMS, E., 92
Waldstein, Louis, selection, 325
Wattles, J. H., 509, 628
Waugh, James A., 467, 649
William J., 670
West, Sr., W. L , 162
Wellner, H., 465
Williams, W. L., 391, 613
Willyoung, L. E., 721
Worth your knowing, 460
Wyoming Valley Veterinary Association, 381 607

"X," 387

